# A dual recurrent neural network model of human-like motion for artificial agents and its evaluation in a VR mirror game turing test

**DOI:** 10.1038/s41598-025-27712-4

**Published:** 2025-11-18

**Authors:** Marius S. Knorr, Jan P. Bremer, Till R. Schneider, Andreas K. Engel, Alexander Maÿe

**Affiliations:** Department of Neurophysiology and Pathophysiology, University Medical Center Hamburg-Eppendorf, 20246 Hamburg, Germany

**Keywords:** Social interaction, Human robot interaction, Mirror game, Sensorimotor contingencies theory, Sensorimotor processing, Social behaviour, Human behaviour, Cognitive neuroscience, Network models

## Abstract

Action-oriented approaches to cognition which emphasize the constitutive role of sensorimotor patterns for perception are gaining importance for the study of cognitive processes in the human brain as well as for endowing artificial agents with cognitive capabilities. It is still debated whether motor-based action-effect contingencies can be extended to social contexts. Here, we investigate the hypothesis that social sensorimotor contingencies (socSMCs) substantially contribute to successful social interaction, and that endowing an artificial agent with socSMCs could make it an interaction partner evaluated like a human. We studied a variant of a Turing test in which human participants had to decide whether they interacted with an artificial agent or another human. To disguise the true nature of the partner, movements were mapped to standardized avatars who interacted in a virtual environment. Depending on individual traits of the participants and the duration of the interaction, in about 74% of instances participants correctly identified the interaction partner. Subjects were less likely to detect an artificial agent the more they focused on the joint task rather than on the partner. Our results suggest that the subjective experience of physical social interaction to a significant extent accrues from basic sensorimotor patterns.

## Introduction

Socially assistive robotics is a rather new field of research that aims at endowing robots with the capacity for acting in socially aware and engaging ways^1^. The different scenarios of human-robot interaction (HRI) can be categorized according to the characteristics of the interaction: The focus can be on physical interaction, for example, with assembly line robots, or it can have a strong social component, e.g., with restaurant waiter robots (Catering Robot Xiaozhi, HRG Zillion Robotics, China), ‘smart’ medicine dispensers (Pillo, Pillo Health, U.S.), museum (Pepper, SoftBank Robotics, Japan) or airport guide robots^2^.

With the physical and cognitive capabilities of socially assistive robots advancing, people may more and more want to integrate them into their daily lives. This requires developing methods for a proper social coupling with the user. Whether an engaging relationship can be built up depends on several aspects, such as the physical presence and appearance of the robot and the shared context with the user^3^. An embodied robot has a greater impact on the user than a virtual agent^1^, which is important when it comes to motivating the user or increasing user compliance with, e.g., robots for the elderly that remind them to take medicine. The relevance of appearance also becomes evident when observing imperfect humanoid robots—either actual robots or poorly executed human modeling and animation in video games: An imperfect resemblance to a human in terms of appearance or facial expressions may appear unsettling or even repulsive. This effect also pertains to the motion profile of a robot^4^, as specific movements underline the appearance. An interaction with a robot which has a likable appearance but rigid movements would still seem odd. In other words, it is not enough to have a natural appearance, but the robot should move in a natural manner, too. Therefore, motion coordination contributes to the experience of interaction.

Between humans, motion coordination occurs both intentionally and unintentionally. In sports which require synchronicity (partner dance, synchronous dive, etc.), agents actively try to synchronize their movements. For achieving truly fluent and effortless interaction, more frequently unintentional synchronization figures in. Unintentional synchronization occurs spontaneously and often cannot be avoided^5^. A well-known example is spontaneous synchronization when an audience is applauding, during walking, or the unintentional posture mirroring of sympathizing partners.

Proper synchronization requires a continuous integration of the partner’s posture (sensory input) and one’s own current posture (motor output). Throughout the interaction, partners observe patterns and dependencies between their own actions and their effects on the other agents. That such action-effect contingencies, entwining the partners in their interaction, give rise to the experiential quality thereof, is a core component of the concept of social sensorimotor contingencies.

Sensorimotor contingencies (SMCs), which basically are dependencies between sensory inputs and motor actions of an agent, have previously been suggested to explain perceptual awareness and (visual) consciousness^6^. The key idea is that action together with the resulting changes in sensory input gives rise to subjective experience of the environment^6^. SMC theory diverges from the traditional view of agent control by a loop of interleaved action and perception processes and instead assigns action a constitutive role for perception.

This notion of SMCs in individual cognition has been extended to social contexts. The subjective experience of a social interaction, accordingly, emerges from patterns in the sensorimotor coupling that develops between the agents. We have suggested the term ‘social sensorimotor contingencies’ (socSMCs) for extending sensorimotor contingency theory to social contexts^7^. Acknowledging the constitutive role of action in social cognition has widespread ramifications for the interpretation of existing findings and future research in fields such as neuroscience, psychology, and human-robot interaction^8^. Here we investigate whether and how this concept may help develop robot control architectures which can make the interaction of a robot with a human feel as natural as possible. Whereas the model ought to implement core ideas from the socSMCs concept, we will not require it to also reflect potential mechanisms in the brain that are involved in social interactions.

The study we present in this article focuses on the coordination aspect in social interactions. Our main research question is whether learning and deploying such action-effect contingencies might be sufficient to establish a proper coupling during social interaction. If socSMCs contribute substantially to successful social interaction, an artificial agent equipped with socSMCs should afford a more human-like interaction. To test this hypothesis, we investigated human-human interactions (HHI) and human-robot interactions (HRI) in a virtual reality (VR) environment. This approach allowed us to endow all agents, no matter whether artificial or biological, with a uniform virtual embodiment. The advantage of uniformity is that participants are not primed by features of the visual appearance of the partner. This ensures that only the sensorimotor experience can contribute to how the interaction and interaction partner are experienced.

For the interaction paradigm, we selected a three-dimensional version of the mirror game^9^. Mirroring is a popular element in joint improvisation techniques and a key component in dance and movement therapy, allowing a person to explore the effects an action has on the interaction partner^10^. The task in this interaction is to reproduce the movements of the partner as precisely as possible. Movements are initiated by the leader and followed by the partner. The roles can be determined by instruction or negotiated between the players. Playing the mirror game requires both interaction partners to continuously adapt to the other player, i.e., continuously integrate sensory information, and update the own hand position. In the original mirror game paradigm^9^, where players used a slider (1d) to synchronize their movements, the social aspect is less pronounced; a gap which we try to overcome by a 3d paradigm. The 3d paradigm with virtual avatars resembles much more a social interaction setting where specific cues (e.g., gestures) lead, often unintentionally, to the execution of a certain action (e.g., reply to the gesture). An oscillator model has been used for controlling a virtual avatar in a 1d mirror game^11^. To validate and improve the model’s performance, the authors suggested to employ a Turing test.

In our study, movements of the avatar in the VR environment were controlled either by data from a system that tracked the movements of the upper body of human players in real-time or, in the case of the artificial agent, from a computational model that had acquired socSMCs. Control of the avatar was switched, on a trial-by-trial basis, between these two sources. Subjects were not aware of these switches. This setup allowed us to investigate participants in HHI or HRI in exactly the same environment.

To assess the success of the model in capturing relevant socSMCs, we monitored the participants’ belief about the nature of the partner. This is a variant of a Turing test for ascertaining whether a machine, in this case a computer system, can show human-like behavior to an extent that makes it indistinguishable from the behavior of a real human. Alan Turing originally established the imitation game in which an interrogator communicates with a male and a female over a teleprinter and has to decide who is male and who is female based on their answers^12^. The male and female could imitate each other to trick the interrogator. Turing’s follow-up question was whether it was possible to replace the male with a computer and still get similar results in terms of the error rate. Over time, the Turing test evolved into a general method for assessing the capability of a machine to imitate human behavior and even serves as the subject of a variety of movies (e.g., Ex Machina, 2014). For the artificial agent, we employed two Long Short-Term Memory (LSTM) networks^13^ that were trained on motion data from several participants. One network was trained to reproduce movements of a human follower (follow-net), whereas the other learned to predict good continuations of a starting movement sequence (lead-net). The outputs of the networks were superimposed and controlled the joint angles of the avatar. This setup had the capacity for acquiring a broad range of motion features which are common across the population. A sensitivity analysis of the follow-net’s inputs provided insights into the acquired socSMCs by revealing the importance that the model assigned to individual limbs of the interaction partner when following movements.

## Results

### Movement characteristics of humans and the artificial agent differ

We analyzed the motion data that were recorded from humans and the artificial agent from three perspectives: (i) movement properties of individuals, (ii) dynamics of the interaction, and (iii) in the humans, relations of movement and interaction parameters with subjective experience.

**Figure 1:**
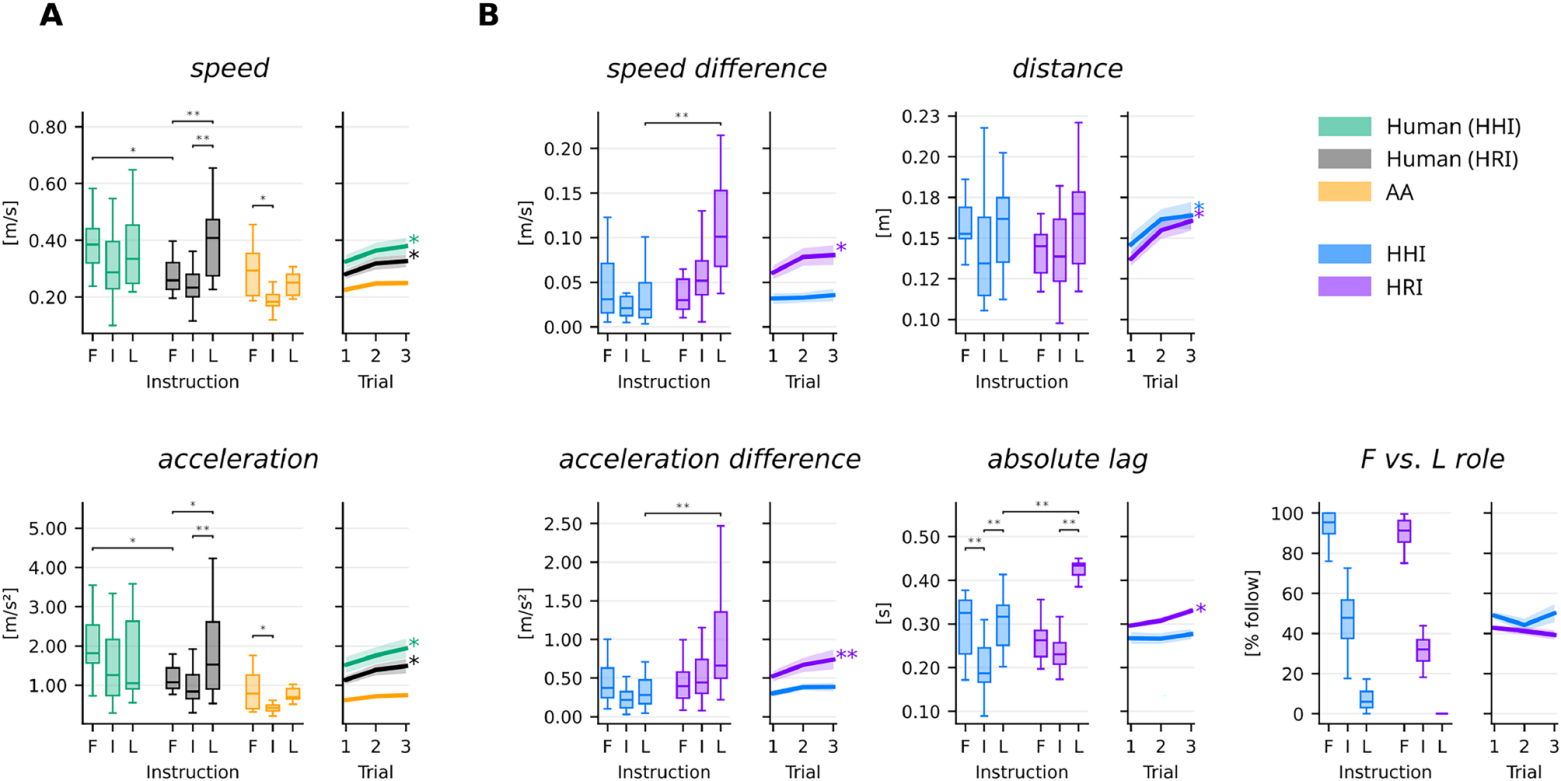
Contrasting kinematic properties of human-human interaction (HHI) and human-robot interaction (HRI). **(A)** Individual motion parameters: mean speed ($\bar{v}$) and mean acceleration ($\bar{a}$) of the human in HHI (green), the human in HRI (grey), and the artificial agent (yellow). The x-axis shows the trial condition (F-follow, I-improvise, L-lead). **(B)** Interaction parameters: differences of mean speed ($\Delta \bar{v}$), difference of mean acceleration ($\Delta \bar{a}$) and distance between the agents’ hands (*d*). The last two panels show the absolute lag ($\hat{t}$) and the average direction of the lag ($r_{LF}$) calculated from the windowed time lagged cross-correlation (WTLCC) between the hand trajectories.

First, we investigated whether the task instruction or the kind of interaction partner had an effect on individual movement speeds. The average speeds per trial were submitted to a two-way repeated-measures ANOVA with the factors instruction (lead, follow, improvisation) and partner type (human, artificial). Both main effects were significant (instruction: $F({2}, {46}) = {10.78}$, $p = {0.0028}$, partner type: $F({1}, {23}) = {14.03}$, $p = {0.021}$). Post-hoc comparisons (Tukey’s Honestly Significant Difference) indicated that the artificial agent moved slower than the humans in all conditions (Fig. 1A). Accordingly, participants moved slower when following the artificial agent compared to when following another human (Tukey’s HSD, $p = {0.0149}$). When mirroring the artificial agent, humans moved faster when instructed to lead compared to the follow and improvisation conditions (improv.: $p = {3.3}\times 10^{-5}$, follow: $p = {0.0002}$). To test the influence of the task instruction on the movement speeds of the artificial agent, we employed a Friedman test and found a significant effect ($\chi ^{2}({2}, {18}) = 21.77$, $p = 3.7\times 10^{-4}$). A Nemenyi post-hoc test showed that the artificial agent moved significantly slower when improvising compared to leading ($p = {0.0258}$) or following ($p = {0.0062}$). Over time, humans increased their movement speed in both HHI ($p = {0.009}$) and HRI ($p = {0.009}$) (Fig. 1A, right panel). The artificial agent showed a similar trend which however did not reach the significance threshold ($p = {0.063}$). Accelerations showed the same pattern.

Next we analyzed the interaction dynamics in terms of the time-averaged speed differences per trial between the partners’ movements. A two-way repeated measures ANOVA revealed significant main effects of instruction ($F({2}, {34}) = {9.98}$, $p = {0.0077}$) and partner type ($F({1}, {17}) = {27.86}$, $p = {0.0012}$). Speed differences were conspicuously larger when the artificial agent followed the movements of the human (Tukey’s HSD, $p = {1.2\times 10^{-5}}$, see Fig. 1B). That is, the human agents could adjust their movement speeds and accelerations better than the artificial agent to their interaction partner. Together with the increasing movement speeds over time, the speed differences also increased, but only when participants interacted with the artificial agent ($p = {0.009}$), and not when interacting with another human ($p = {0.28}$) (Fig. 1B, right panel).

Unlike speed and speed difference, we could not establish an effect of task instruction and interaction partner on the distance between the agent’s hands (all $p > {0.9}$). However, distances increased across trials (HHI: $p = {0.009}$, HRI: $p = {0.002}$), which is likely a consequence of the increasing movement speeds.

Mirroring the movements of a leader incurs a small delay in the follower which can be evaluated by the windowed time-lag cross-correlation (WTLCC, Fig. 1B). Using a repeated-measures ANOVA, we found that the instruction and the partner type had a significant effect on this lag (instruction: $F({2}, {34}) = {48.33}$, $p = 1.1\times 10^{-10}$; partner type: $F({1}, {17}) = {13.79}$, $p = {1.7\times 10^{-3}}$). Post-hoc comparisons revealed that in HHI, the lag during improvisation was significantly smaller than in the follow ($p = {1.05\times 10^{-4}}$) and lead conditions ($p = {1.3\times 10^{-5}}$). In HRI, the lag during improvisation was smaller than in the lead condition ($p = {2.3\times 10^{-14}}$), but not when following ($p = {0.7265}$). Notably, the lag at which the artificial agent followed human movements was significantly larger and less variable than when humans followed human movements ($p = {1.74\times 10^{-8}}$). In HRI, the lag showed a rising trend over time ($p = {0.009}$).

In addition to the lag, WTLCC also yields the direction on the time axis in which one trajectory has to be shifted to maximize the correlation with the other. Integrating this direction information (sign of $\hat{t}$) over a trial yields a measure for the time an agent was leading or following the partner. This ratio was close to 0% / 100% in the lead / follow conditions, respectively, confirming that the agents indeed abided by the instructions (Fig. 1B, last panel). Ratios around 50% indicate that in human joint improvisation, the intervals of leading and following were balanced. When improvising with the artificial agent, however, humans had a tendency to lead more often than to follow (one-sample t-test against 50%: $t({17}) = {-4.78}$, $p = {0.0001}$).

### Humans can’t reliably distinguish between human and artificial agents

To assess how well the artificial agent mimicked human mirror-game behavior, we analyzed the responses of the participants after each trial, specifically to the question whether they believed that their interaction partner was human or artificial. Despite the objective differences in the movement characteristics of human and artificial agents, participants failed to detect the artificial agent in 26.9% of the trials, mistaking it for a human (false negative rate (FNR) across all conditions (Table 1)). This type of misjudgment occurred most often in short trials, in particular when leading or following. Vice versa, participants took a human partner as artificial at a similar rate (25.8%, false positive rate (FPR)), mostly when instructed to lead and in long trials. Participants most reliably detected the human partner when following movements in short trials (FPR = 10%). Due to the low number of trials, we could not establish a difference between short and long trials when considering the conditions individually. However, a significant effect of short vs. long trials on the FPR (16.7% vs. 28.3%) was found when considering trials from all conditions ($p = {0.0255}$).

**Table 1:** Mean classification errors across participants in the Turing test. HRI and HHI trials were balanced. The false negative rate (FNR) indicates how often an HRI was misclassified; the false positive rate (FPR) indicates how often an HHI was misclassified. Note that with an artificial agent which perfectly emulates human movements, participants could only guess the nature of the interaction partner (chance level: 50%). Numbers in parentheses are not significantly different from a random guess.

	Overall (%)	Follow (%)	Improvisation (%)	Lead (%)
False negative rate	27	27	25	30
False positive rate	26	21	26	29
Accuracy	74	76	75	70
False negative rate (long trials)	25	25	24	28
False positive rate (long trials)	28	24	28	32
Accuracy (long trials)	73	76	75	70
False negative rate (short trials)	30	(33)	(25)	(35)
False positive rate (short trials)	17	10	22	23
Accuracy (short trials)	76	78	77	71

**Figure 2:**
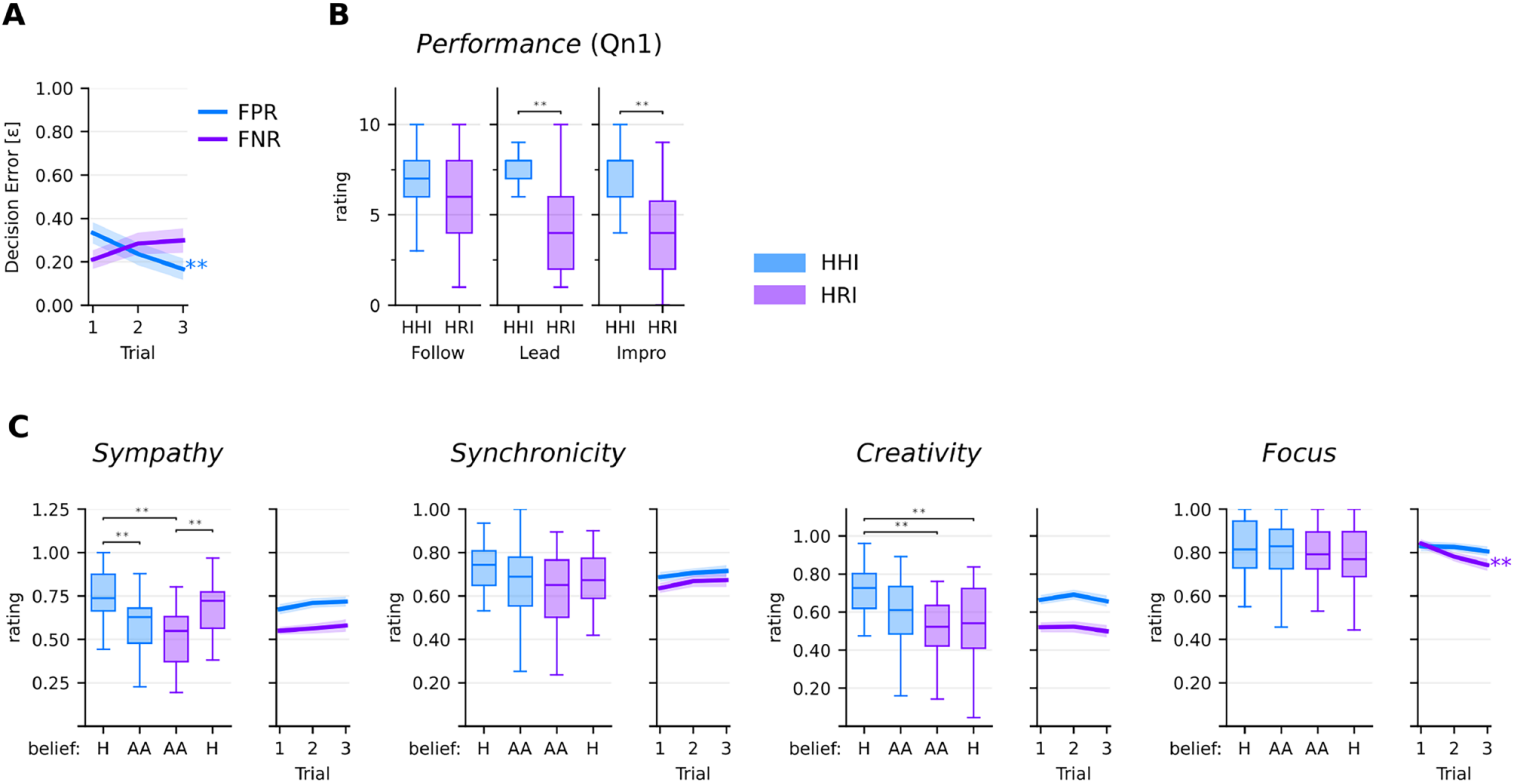
**(A)** Partner identification errors in HHI and HRI trials, respectively. **(B)** Subjective performance evaluation at the end of the experiment (Qn1). The instruction on the x-axis is the partner instruction. **(C)** Subjective experience ratings after each trial. **$p<0.01$, AA = artificial agent, H = human.

Participants improved their ability to identify a human partner throughout the experiment (Fig. 2A). Their ability to detect the artificial agent, in contrast, decreased with time and was lowest in the short trials at the end of the experiment. To shed light on the factors that contribute to errors in identifying the partner type, we fit a linear mixed-effects model with the decision error $\varepsilon$ as the dependent variable, using movement parameters as fixed effects and trial and participant as random effects (Table 2). Fixed effects of acceleration difference ($\Delta \bar{a}$, $p = {0.011}$) and instruction (leading, $p = {0.010}$) were observed, suggesting that participants exploited the larger speed and acceleration differences when leading in an interaction with the artificial agent to detect it (see Fig. 1B).

**Table 2:** Linear mixed-effects model result for the decision error $\varepsilon$ (dependent variable).

Predictor	Estimate ($\beta$)	Std. Error (SE)	z-value	*p*-value	Sig.
(Intercept)	-0.283	0.782	-0.362	0.717	
Condition: improvisation	0.239	0.316	0.756	0.450	
Condition: leading	0.821	0.318	2.585	0.010	**
mean speed ($\bar{v}$)	0.600	2.976	0.201	0.840	
mean acceleration ($\bar{a}$)	0.288	0.339	0.851	0.395	
speed difference ($\Delta \bar{v}$)	2.714	4.973	0.546	0.585	
acceleration difference ($\Delta \bar{a}$)	-1.486	0.582	-2.555	0.011	*
distance (*d*)	-7.361	4.955	-1.486	0.137	

### Overall appreciation of the interaction

After completing the experiment, we asked participants to evaluate the overall performance of the partners on a discrete scale from 0 (‘bad’) to 10 (‘good’, Qn1, Fig. 2B). These ratings were separate from the ratings after each trial and aimed to get the general attitude towards the interaction partners under different conditions. A two-way ANOVA with factors instruction (lead, follow, improvisation) and partner type showed significant main effects for both factors ($F({2}, {74}) = {5.5}$, $p = {0.006}$, partner: $F({1}, {37}) = {54.79}$, $p = {8.3\times 10^{-9}}$). Follow-up pairwise contrasts using Tukey’s HSD showed that performance scores were different for the artificial agent and humans in the lead ($MD = {3.03}$, $p = {3.4\times 10^{-8}}$) and improvisation ($MD = {3.23}$, $p = {7.7\times 10^{-9}}$) conditions.

We also assessed the perceived ratio between HRI and HHI trials (Qn2, scale: 0 ‘always artificial agent’ to 10 ‘always human’). In fact, the number of HRI and HHI trials was balanced ($M = {5.0}$), but except for one participant, the responses were biased toward more HRI trials than HHI ($M = {4.13}$, $SD = {1.28}$, one-sample t-test against 5: $t({37}) = {-4.12}$, $p = {0.0012}$).

In addition to the ratio between perceived partner types, we also interrogated participants about the perceived ratio between episodes of leading and following during improvisation trials (Qn3, scale: 0 ‘always follower’ to 10 ‘always leader’). Participants had the impression that the times of leading and following were balanced, independently of the partner type (HHI: $M = {5.24}$, $SD = {1.1}$, one-sample t-test against 5: $t({37}) = {1.32}$, $p = {1.0}$, HRI: $M = {5.39}$, $SD = {2.53}$, one-sample t-test against 5: $t({37}) = {0.96}$, $p = {1.0}$). This was surprising, because objectively the artificial agent was mostly following; therefore, ratings should have been lower in HRI.

The participants’ experience of improvisation in HRI was more one of alternating leading and following than of co-leadership (Qn4, $M = {3.76}$, $SD = {2.5}$, one-sample t-test against 5: $t({37}) = {-3.01}$, $p = {0.027}$). In HHI, in contrast, the experience was more balanced ($M = {4.76}$, $SD = {2.48}$, one-sample t-test against 5: $t({37}) = {-0.58}$, $p = {1.0}$).

### Ratings of subjective experience

After each trial, participants were asked to rate the sympathy and synchronicity with the partner, the creativity of the movements, and their own focus (Fig. 2C). To test whether the ratings were influenced by the belief in the nature of the interaction partner, we employed four one-way ANOVAs with the respective rating as the dependent variable and the four elements of the confusion matrix as the independent variable. Except for the focus, the main effects were significant for all experience ratings, i.e., sympathy ($F({3}, {78}) = {24.3}$, $p = {1.3\times 10^{-10}}$), synchronicity ($F({3}, {78}) = {5.5}$, $p = {0.0066}$), creativity ($F({3}, {78}) = {13.2}$, $p = {1.8\times 10^{-6}}$). Sympathy ratings dropped when participants believed to interact with the artificial agent although the partner was human (Tukey’s HSD, $p = {0.0017}$). Conversely, they rated sympathy higher when they believed the partner was human when they interacted with the artificial agent instead (Tukey’s HSD, $p = {0.0006}$). Unlike sympathy, the experiences of synchronicity and creativity were not modulated by the belief about the interaction partner.

Finally, we tested whether the subjective experience was shared between partners. Pearson’s correlation analysis did not reveal evidence of reciprocity (all $p > {0.05}$), though. This observation is in line with the findings of a previous study in which the partner did not reflect the feeling of ’good collaboration’ in a joint action task^14^.

Whereas the dyadic experience dimensions did not show a trend over the duration of the experiment, ratings of the individual focus level seemed to decline over time, but only for HRI trials (Fig. 2C).

### Action-effect contingencies between human and artificial agents

**Figure 3:**
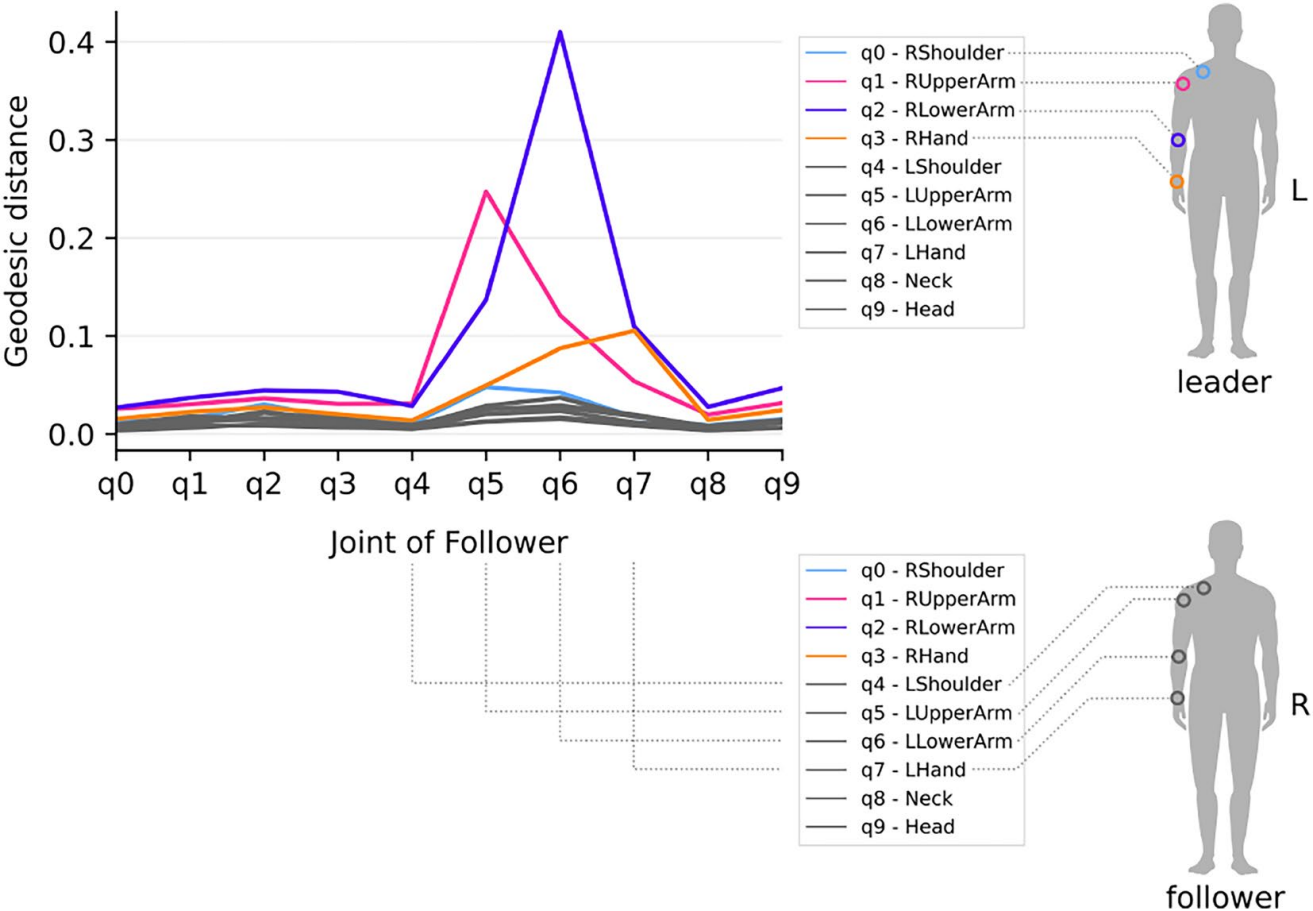
Modeling sensorimotor coupling between partners by comparing the output of the follow-net in terms of geodesic distance for two almost identical motion sequences, in one of which the orientation of joint q was perturbed.

We analyzed mutual action-effect patterns in the movements by assessing the feature sensitivity of the follow-net as a model of a human follower. The features, i.e., the joint orientations of the leader’s arm and shoulder (see legend in Fig. 3), were perturbed, and the resulting change in the output of the follow-net was measured by the geodesic distance. A larger distance indicates a stronger influence of a leader joint (colored line) on a specific follower joint (x-axis in Fig. 3). This perturbation analysis shows that the right lower arm of the human had the largest influence on the corresponding (left) lower arm of the artificial agent (q2 $\xrightarrow {}$ q6). The same holds for the upper arm (q3 $\xrightarrow {}$ q7) and the hand (q1 $\xrightarrow {}$ q5). Changes of the lower arm rotation of the leader had the strongest impact on the follower (q2 curve is higher), followed by the upper arm (q1) and the hand (q3). The right shoulder did not seem to play a role, as its movements were subtle. In general, joints did not only influence the corresponding but also the adjacent joints of the partner. The influence of the upper arm on the hand (q1 $\xrightarrow {}$ q7) was small. Likewise, the influence of the hand joint on the respective other agent’s hand was small, indicating that mirroring the hand rotation often was neglected and that, rather, the wrist’s broad position was important.

## Discussion

We have introduced a computational model for acquiring socSMCs and employing them to control an artificial agent. The behavior of the model is generated by mixing the outputs of two networks which have been trained to create and follow movements, respectively. Both networks were trained on kinematic data from human upper body movements. Due to their recurrent structure, the networks naturally integrate previous motion and (in the case of the follow-net) motion from the leader. The architecture does not build on separate modules for perceiving or representing the interaction partner and for motor planning and execution, but memorizes and recalls composites of body poses and arm movements instead, which we consider as a computational model of sensorimotor contingencies. Notably, these contingencies not only encompass the agent itself or the partner but also capture patterns of their interaction.

The function of the follow-net seems to bear some resemblance to the so-called mirror neurons that have been found in the brains of macaques^15^ and the human mirror system^16^ in at least two respects: First, its activity reflects the movements of the partner, and it controls the movements of the artificial agent in the follow condition. And second, it acquires this function through sensorimotor learning^17,18^. This resemblance, however, may emerge from the specific task that we studied here, the mirror game. The role of a mirror system in the lead and improvisation conditions is much less obvious. From an action-oriented perspective on social cognition, subjective experience is constituted through the sensorimotor patterns that drive a social interaction. The mirror system may be a component of the mechanisms that allow us to engage in social interactions, but it is very likely not the sole origin of the social experience^19^.

The subjective experience of humans in the interaction with this agent was evaluated in terms of sympathy, synchronicity, and creativity. In a Turing-test-style experiment, we investigated in how much the socSMC-controlled agent resembled a physical interaction between humans. Virtual reality was used to conceal the true nature of the partner (human or artificial) and standardize all features of visual appearances. Therefore, the interaction was not affected by expectations towards the partner, and the subjective experience was shaped solely on the basis of sensorimotor interaction patterns.

One characteristic of HHI is the unintentional coordination that emerges spontaneously. This coordination leads to a feeling of togetherness, also sometimes referred to as ‘being in the zone’^20^. While some interactions take place in an intuitive way, others lack a silent, mutual understanding of the partners. In particular, compassionate interaction partners can show flawless unintentional coordination. Yet, sympathy may occur just because the interaction happened intuitively. That is, the sensorimotor basis of interaction may contribute, at least to a certain extent, to the subjective experience. Our results suggest that people feel more sympathy with the partner when they believe to interact with another human, even if this belief is false. Therefore, endowing robots with the ability to adjust their movement patterns to the human partner holds great potential for making HRI feel natural.

Contrary to the findings of the original one-dimensional mirror game paradigm, in which two sliders mirrored the position along a line^9^, in our study the motion trajectory of the follower did not dither around the trajectory of the leader. This is not surprising, since 3d mirroring involved the coarse processing of spatial limb position instead of the processing of a slider position with high spatial resolution in the 1d version. In one dimension, position errors and inconsistencies are easier to notice, allowing the reactive partner to continuously adjust its position, which can lead to transient oscillations around the active partner’s location. However, in our experiment, the participant’s hand was out of sight most of the time, as the VR has a restricted field of view ($<110^{\circ }$). Therefore, participants had to rely on proprioception and less on visual control.

In 1950, Turing predicted that in about 50 years’ time, an interrogator would identify the partner in the imitation game in no more than 70% of the interactions after 5 minutes of questioning^12^. A recent chat-based Turing-test study found that state-of-the-art models (GPT-4o) were judged to be human in up to 77% of conversations after 5 minutes of interaction, suggesting that AI systems can convincingly mimic human behavior in short conversations^21^. In contrast to this verbal interaction, another experiment involving communication through movements of abstract 2D shapes achieved an identification accuracy of 74%^22^. Our Turing test results indicate that the computational model was able to learn at least some of the sensorimotor patterns that are crucial for a natural interaction with another human. In about 74% of the interactions, the participants correctly identified the partner. Whereas two participants always correctly detected the type of interaction partner, six misclassified most interactions. We employed a linear mixed-effects model to elucidate the effects of the various movement parameters on the decision error. This analysis revealed that the acceleration difference and the condition (leading/following) had a significant effect on the decision error. We hypothesize that participants noticed that the artificial agent was unable to maintain rapid velocity changes and used this in their decision. This could also explain why movement variations in terms of acceleration increased over the course of the experiment (Fig. 1). Basically, participants regularly challenged the agent, raising the requirements for the control mechanism to mirror the movements easily beyond the level that the proposed model could achieve. Therefore, an average detection accuracy of 74% is considered a successful test result which is similar to previous Turing test paradigms. Interestingly, most of the participants did not become aware that they followed such a strategy to identify the interaction partner. Only a few of them stated that they could identify an HRI by focusing on certain unnatural movements, especially in periods in which the artificial agent led the movements (follow task). The majority instead declared that they could feel that there was just something ‘not right’, without explaining in detail how they arrived at this impression.

Early trials and late trials strongly differed in terms of identification accuracy in HHI vs. HRI: The identification accuracy in HHI increased over time, whereas the accuracy in HRI showed the opposite trend. This might have several reasons. In the first trials, we observed a higher accuracy in recognizing the type of the interaction partner. Participants were still under the impression of the task instruction and therefore less inclined to focus on how the interaction felt. They rather focused on inconsistencies and revealing errors, even violations of anatomical constraints which would imply an interaction with a robot. That is, HRI could easily be identified (low FNR), while ‘imperfect’ HHI would be misclassified (high FPR). In later trials, the participants’ ability to focus on the task decreased. Thus, there were less resources for motion error detection, and the interaction felt increasingly intuitive. Participants reported to be more ‘in the flow’ in later trials. The experience of interaction now became more essential than the task. At the same time, this may have reduced their ability to detect movement patterns which are apt to reveal an interaction with the artificial agent. This would explain why, towards the end of the experiment, HRI classification degraded (high FNR). We hypothesize that as long as participants are not actively looking for mismatches between their expectations and the actual interaction, a computer can in fact acquire relevant socSMCs for an appreciable interaction. In this case, the interaction would not be distinguishable from HHI.

Taking these results together, we suggest that there is a type of socSMCs which is particularly relevant for establishing social coupling and which we call check-SMCs^7,8^. Check-SMCs capture a unidirectional information transfer from the observed to the observer. In this respect, check-SMCs seem to bear a relation to the concept of motor contagion. Motor contagion or motor resonance describes the inter-subject adaptation of the observer’s motor repertoire upon watching movements of an agent^23^. Motor contagion is induced not only when observing humans, but also when observing humanoid robots as long as anatomical constraints (i.e., non-biological velocities) are not violated^23^. We suggest that the reduced appearance of the humanoid avatars in our virtual environment still allowed the partners to establish a coupling which may exhibit motor contagion.

The analysis of motion parameters revealed that socSMCs are dynamically shaped for optimizing task performance. For example, the mean speed increased over time, whereas the speed difference between the hands was kept steady in HHI, but increased during HRI. Faster motion accompanied by stable speed differences suggests that the interaction partners had become acquainted with each other and had embraced each other’s motion repertoire. An interesting observation is that the lag between the partners’ movements was substantially smaller during improvisation than in the lead and follow conditions. This suggests that joint improvisation involved better synchronization and hence a tighter coupling than the instructed roles of a leader or a follower.

We conclude by discussing some limitations of the artificial-agent controller’s design. This design used two neural networks to function as a leader, a follower, or, by blending the activity of both, to improvise movements in the mirror game. One limitation concerns the dynamics of the lead-net. Participants identified the artificial agent more accurately when the agent was leading. Its movements were smaller than average, and they lacked cues that could be used to anticipate upcoming movements by the follower. In addition, participants reported a lack of visible intention of the artificial agent. Moreover, the lead-net only slightly regulated motion characteristics of the artificial agent, e.g., motion speed in response to the follower, to maintain a synchronous yet creative motion-based interaction. In other words, it generated movements in an open-loop manner, without considering feedback from the partner. To equip the lead-net with a certain awareness for the human follower, we superimposed a weak (10%) follow behavior. However, the gross behavior was generated by replaying a pre-synthesized, yet unpredictable sequence of motion. Experiments in which participants had to synchronously tap their fingers revealed that predictability and reactivity can partially compensate each other^24^. Future studies should therefore employ a lead-net that generates movements instantaneously rather than playing pre-computed sequences.

The follow-net performed best in terms of imitating a human, as it was the reactive part with a lower influence on the interaction. Its main goal was to mirror the movements as precisely as possible. Yet, it could be uncovered by movements that have a highly symbolic value for humans like, for example, waving. Hand waving is a canonical means of attracting the attention of other humans. Obviously, the follow-net does not have this understanding. In such instances, it did not anticipate the repetitive nature of the waving movement but, rather, followed with the exact same delay. This caused an impression of a quite artificial waving which makes it easy for the human to distinguish HRI from HHI. By adding a predictive component, the model could become faster in mirroring repetitive movements and more responsive when following movements. This may likely make the artificial agent even more indistinguishable from a human. In view of the relatively high misclassification rate, an improved model should be able to take the Turing test to the chance level.

## Methods

### Procedure

The experiment consisted of two sessions: The training session and the Turing test session. In this paper, we analyze data from the Turing test; results from the training session will be reported in a separate publication^25^.

#### Training session

We invited 48 participants in total, aged 19 – 33 years ($M = {22.79}$, $SD = {2.73}$; 28 females) without any record of psychiatric or neurological disorders. Most of them were medical students. Wearers of glasses were asked to put on contact lenses to avoid interference with the VR-goggles. Participants were grouped in 24 dyads. The two partners were located in different rooms, and they did not meet before the end of the experiment, nor did they obtain any information about their partner. They received detailed instructions about the procedure both orally and in written form. The study was approved by the ethics committee of the medical association of the city of Hamburg, Germany, and strictly adhered to the Declaration of Helsinki and the principles of good scientific practice. All participants provided written informed consent.

**Figure 4:**
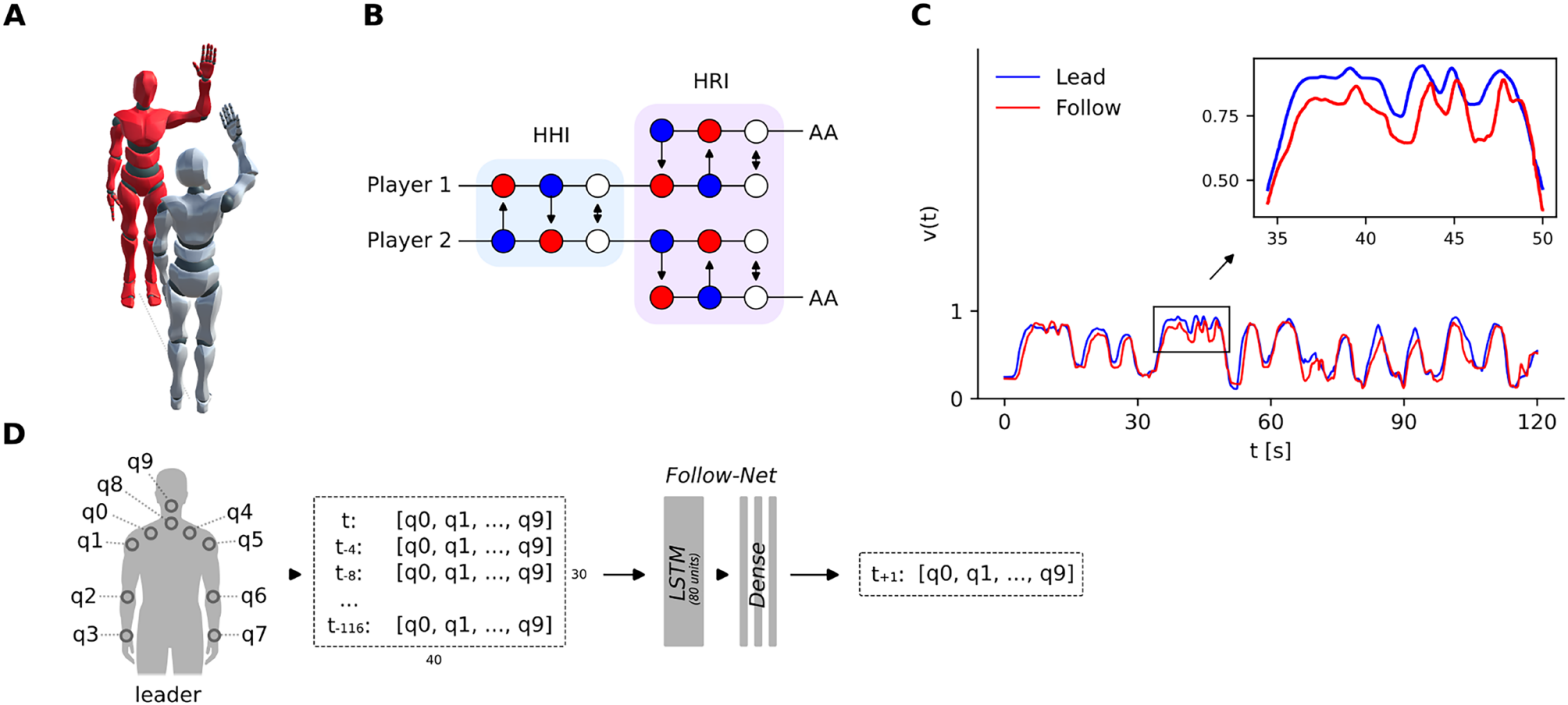
**(A)** Visual representation of the avatars performing the mirror game. The 3d visualization was generated using Unity 2022.3.30f1 (https://unity.com). **(B)** The experiment employed six conditions: Leading (blue), following (red), and improvisation (white) with either a human interaction partner (human-human interaction, HHI) or an artificial agent (human-robot interaction, HRI). The true nature of the interaction partner was unknown to the participants and had to be identified in a Turing test-like setup. In addition, participants had to rate the subjective experience in terms of synchronicity, sympathy, creativity and focus after each trial. **(C)** Typical profile of hand speed in 3d during HHI L/F. The red curve follows the blue with a delay. **(D)** The follow-net outputs joint orientations for the next frame ($t+1$) based on the most recent 1.3 s (every fourth of 120 frames = 30 frames) of the partner’s upper body motion. Upper body motion is represented by quaternion orientations of 10 joints. LSTM cells provided output to three feed-forward layers.

Before preparing the players for the experiment, they were requested to fill a set of questionnaires (see section on Questionnaires and Ratings below). Then they were equipped with the motion tracking system. After a short calibration period, they were also fitted with the VR goggles. Before the actual experiment started, there was a short period of checking the functionality and calibration of the VR system. This also helped the participants to accommodate to the new environment. As soon as both participants were ready, the experiment started. Participants were instructed to synchronize their arm movements with the avatar standing in front of them in the virtual setup (Fig. 4A), analogous to a mirror. There was no restriction concerning the characteristics of arm movement. Participants were however told to only focus on their active arm (right for player 1, left for player 2) and to try to avoid eccentric and/or revealing movements of the remaining limbs. Before each trial, participants were instructed to take one of three different roles. They would either lead, follow or improvise arm movements. The leader created arm movements that the follower adopted using the corresponding arm. The improvisation task did not involve a designated leader or follower role, but movements had to be synchronous. After each trial, participants rated the interaction in terms of mental focus, synchronicity, creativity, and sympathy on a continuous scale between zero (‘none’) and one (‘perfect’). The experiment consisted of 15 trials, each lasting 2 minutes. The order of conditions was adapted from Noy^9^: FLI - IFL - FLI - FLI - ILF with player 1 following (F), player 1 leading (L) and improvisation (I). The whole session took approximately 40 minutes. After completing the experiment, we interviewed the participants about their subjective experience during the interaction paradigm and the different conditions.

#### Turing test

Participants who conducted the training session were invited 4 to 6 weeks later to perform the Turing test. They were randomly paired with a different partner. Thirty-eight participants (aged 19 – 33; $M = {22.76}$, $SD = {2.66}$; 21 females) were tested.

In this part of the experiment, participants were also requested to perform the mirror game, but this time we deployed an artificial agent that was trained on motion data gathered from the first session to act as an alternative interaction partner for the human participants and to constitute the HRI condition. All trials from the training session were used to develop the neural networks. Whether participants interacted with each other or with the artificial agent (Fig. 4B), respectively, was randomly chosen beforehand by a simple algorithm which ensured that there were no more than 3 successive trials with the same interaction partner and that interactions with the human and the agent were equally frequent. Participants were not informed with whom they currently interacted but had to decide after each trial (forced choice) and to declare how confident they were with their decision (continuous scale between 0 ‘I don’t know‘ and 1 ‘I am certain’). Confidence ratings did not yield additional insights.

One experiment consisted of 12 long trials (2 min) followed by 6 short trials (1 min). This yielded three trials per condition (L/F/I), two long and one short. We cut the duration of the last trials short as we wanted to decrease the time in the VR and avoid motion sickness, yet have another set of iterations for all three conditions with both interaction partners. Moreover, we aimed to assess the influence of trial duration, as we hypothesized that longer trials lead to higher detection accuracy. The whole experiment took approximately 40 minutes screen time. After completing the experiment, participants were interviewed about their experience (Table 3).

### Hardware setup

Motion was tracked in real-time using two Xsens Motion Tracking Systems (Mtw Awinda, Xsens, The Netherlands) with a 60 Hz sampling rate. Eleven sensors were put on different body parts: unilaterally on the head, pelvis, and sternum, and bilaterally on the shoulders, hands, and upper and lower arms. Two identical virtual environments were created in Unity (Unity Technologies, U.S.), synchronized via TCP sockets. The environments were screened on HTC Vive Pro (HTC/Valve, Taiwan/U.S.) VR-goggles.

### Artificial agent

The virtual agent was controlled by two different neural networks, subsequently referred to as lead-net and follow-net. Both nets were trained on motion information gathered from the participants in the first session.

#### Pre-processing of motion information

In the training session, we collected motion data from 48 participants interacting in all three conditions. The hand speeds of an exemplary leading-following sequence are shown in Fig. 4C. The motion data consisted of 17 rotations describing the relative orientation of every joint. The rotations were stored as quaternions. Quaternions are 4d representations (4 values: *w*, *x*, *y*, *z*) of a rotation in 3d space. Compared to Euler angles, which describe the angle in degrees around all three axes, quaternions have several advantages in the context of interpolating and avoiding gimbal lock (loss of degree of freedom due to two axes being parallel). In addition, there are only two representations of a single orientation (Q and -Q) in contrast to potentially infinite representations in Euler angles $(0^{\circ } = 360^{\circ } = 720^{\circ })$. To provide an adequate input representation for the neural network, we normalized quaternions and reversed their sign if the *w*-value—the first value of a quaternion—was negative. This operation guaranteed a standardized length (uniformity) and constant *w*-value-margin between zero and one. We pre-processed the dataset by converting (mirroring) all active right arms to the left side. After flipping the limbs across the x-axis, the quaternions had also to be adjusted by reversing the sign of their *y* and *z* values. As the neural network was handling motion data only from and for the left arm, the input data from player 1 (right-handed) as well as the network’s output data for player 2 had to be flipped. This allowed us to use the same network regardless of whether the agent had to move or follow with the left or right arm.

#### Neural networks

The artificial agent learned the socSMCs of the mirror game using Long-Short-Term-Memory (LSTM)^13^ neural networks. They were implemented in Python 3.6 with Keras^26^. The follow-net (Fig. 4D) and lead-net had different architectures. The input to the follow-net were the upper body joint orientations of the other player, and the output were the joint angles that generated the movements of the avatar. It predicted the next frame on every fourth of the previous 120 frames in real time. With a locked frame rate of 90 fps, the recent 1.3 seconds would be considered. One prediction took about 3 milliseconds on a GPU (RTX 2080, NVIDIA, U.S.). We trained the follow-net for 15 epochs using the rmsprop optimizer, an initial learning rate of ${1\times 10^{-3}}$ and the mean squared error as the loss function.

The lead-net generated motion *de novo*. We addressed the problem of motion generation as a sequence-to-sequence prediction problem where a ground-truth input sequence of the training data is used to calculate an output sequence. The output was recursively fed back to the network input to generate continuous motion.

Gaussian noise was added during training as a regularization technique. For the experiment, the lead-net was initialized with randomly sampled starting sequences from the training data. The output was recursively fed back to the network 15 times to generate 150 seconds of motion, of which we used the first 120 seconds for the long trials.

We considered joint improvisation as a process of continuously switching the leading and following roles between the partners. We modeled this behavior by superimposing the outputs of the lead-and follow-nets weighted by a role-switching coefficient. This coefficient, which can be either 0 or 1, is determined every 4 seconds, with about 50% probability for a role change. To reinforce a balanced alternation, we gradually increased the probability (by 1%) of becoming leader when following dominated the previous seconds. A common strategy for people to change from following to leading was to induce obvious errors and thereby summon the interaction partner to relinquish leadership and to maintain synchronicity of the movements. Therefore, the agent switched to follow mode once the distance between the hands (*d*) exceeded a threshold of 0.3 units in Unity.

#### Joint control by lead- and follow-net

To establish a smooth interpolation between both networks, we used spherical linear interpolation of quaternions (SLERP^27^). SLERP interpolates between two normalized quaternions $q_1$ and $q_2$ along the shortest path of an arc (‘geodesics’) on the interval $w=[0,1]$:

$$\begin{aligned} q(w) = q_1\exp \left( w\log \left( q_1^{-1}q_2\right) \right) \end{aligned}$$

Unlike linear interpolation, the angular velocity remains constant while interpolation steps are non-equidistant. That is, the approach of spherical linear interpolation is more suitable than linear interpolation for natural rotations in 3d space. Since we did not want the follower to stand completely (unnaturally) still, which would have happened if the leader did not change the arm position, we used SLERP to integrate subtle continuous motion generated by the lead-net when the artificial agent was following (10% leading, 90% following; e.g. $w = {0.1}$). Vice versa, the artificial leading had a subtle ‘following’ component (90% leading, 10% following; e.g. $w = {0.9}$) to imitate a ‘good’ leader which would even adapt to the follower (e.g., wait for the follower to catch up).

### Data analysis

We recorded 171 HHI trials from human-human dyads and, as each participant also interacted with the artificial agent, 341 HRI trials. We had to exclude 12 HHI trials and 18 HRI trials resulting from a freezing of the Unity engine or the VR system. As a consequence of a strongly fluctuating sampling rate in one of the motion tracking devices, we could not analyze the HRI trials from the player acting with the left hand (n=171). That is, 159 HHI trials and 162 HRI trials were considered for analysis.

#### Turing test

Participants were requested to make a binary forced choice decision $\tilde{s} \in \{{0},{1}\}$ about the nature of the interaction partner. The decision error $\varepsilon = \left| s - \tilde{s}\right|$, where *s* is the true type of interaction partner (0: human, 1: artificial agent), is a binary value indicating whether or not the decision was correct: For a correct classification $\varepsilon = {0}$, for an erroneous classification $\varepsilon = {1}$. The false negative and false positive rate were calculated as:

$$FNR=\frac{1}{N_{HRI}}{\sum _{i=1}^{N_{HRI}}\varepsilon (i)}$$

$$FPR=\frac{1}{N_{HHI}}{\sum _{i=1}^{N_{HHI}}\varepsilon (i)}$$

These rates range between 0% (no errors) and 100% (all decisions are wrong). If an artificial agent is indistinguishable from a human, error rates would be at the chance level (50%).

#### Motion parameters

Motion parameters were calculated based on both players’ hand positions given by ${\bf s}(t) = (x(t), y(t), z(t))$ in Unity’s coordinate space. Jitter was reduced by applying a low-pass filter with a 4 Hz cutoff. We calculated the instantaneous velocity $\textbf{v}(t) = {\bf s}(t) - {\bf s}(t+\Delta t)$, where $\Delta t$ is the fixed sample rate of 90 fps. For the analyses we used the norm of the velocity vector (speed) $v(t) = \left\| \textbf{v}(t) \right\|$ in 3d space. For comparing trials, we used the average speed $\bar{v}=\frac{1}{T} \sum _{t=1}^T v(t)$ over the trial with *T* samples. The difference between the mean movement speeds of both interaction partners was calculated by $\Delta \bar{v} = |\bar{v}_1 - \bar{v}_2|$.

Acceleration was calculated by $\textbf{a}(t)=\textbf{v}(t) - \textbf{v}(t+\Delta t)$, the mean acceleration by $\bar{a}=\frac{1}{T}\sum _{t=1}^T \left\| \textbf{a}(t) \right\|$, and the acceleration difference by $\Delta \bar{a} = |\bar{a}_1 - \bar{a}_2|$.

The Euclidean distance *d*(*t*) between the hands of both players was defined as:

$$\begin{aligned} d(t)=\sqrt{(x_1(t)-x_2(t))^2+(y_1(t)-y_2(t))^2} \end{aligned}$$

The trial-averaged hand distance was calculated as $d =1/T\sum _{t=1}^Td(t)$.

We compared both individual and joint motion parameters between the training session and the Turing session by averaging across trials and applying independent t-tests for all three instructions (lead, follow, improvisation) respectively. No significant differences were found, neither in the individual nor in the joint motion parameters, between the two sessions.

A common tool to quantify the kinematics of leader/follower relationships between two time series is the windowed time-lag cross-correlation (WTLCC). It determines the lag that yields the highest correlation between two data windows. We used the speed profiles from both partners, because it combines the information from all three dimensions and turned out to be more robust compared to the positions. We set the window size to 300 frames, shifted windows by a step size of 50 frames and varied the lag in the range of $\pm 90$ frames (corresponding to $\pm 1$ s). We calculated the average of the absolute lags with the maximum correlation in each window *i* across a trial by:

$$\hat{t} = \frac{1}{W}\sum _{i=1}^{W} \left| \textrm{WTLCC}(i)\right|$$

Here, *W* is the number of windows in the respective trial. The measure $\hat{t}$ reflects the overall movement coordination independently of the assigned roles (leader / follower). Larger lags occur when players have slower reaction times, whereas smaller lags indicate tight coupling and good synchronization. In addition to the reaction times, we also evaluated the relative duration of episodes of following and leading within a trial by considering the sign of $\textrm{WTLCC}(i)$:

$$r_{LF} = \frac{1}{2}\left( 1 + \frac{1}{W}\sum _{i=1}^{W}\textrm{sgn}(\textrm{WTLCC}(i))\right) \cdot 100\%$$

When instructed to follow, $r_{LF}$ should approach 100%, whereas it should approach 0% when leading.

#### Statistical analyses

To evaluate the difference between the artificial and human players w.r.t. individual and joint motion parameters (Fig. 1), we applied a factorial design using repeated-measures ANOVAs in the Statsmodels Python package. In all analyses, dependent variables belonging to the same factorial cell were averaged across trials. An experiment had 18 trials, two types of interaction partners (human or artificial), and 3 task instructions (lead, follow, improvisation), resulting in 3 trials for each combination (factorial cell). For motion parameters, we employed a two-way ANOVA for the human player with main effects partner (human, artificial) and instruction (lead, follow, improvisation). For motion parameters of the artificial agent, we employed a Friedman test with the factor instruction, because there was only one possible interaction partner and the normality assumption was not met (Shapiro–Wilk test). Data were also examined for homogeneity of variance (Levene’s test). Assumptions were almost always met. If they were not met, QQ plots showed a slight deviation at the tails, confirming normality nevertheless. All reported p-valued were adjusted for multiple comparisons using the Bonferroni method, i.e. multiplying the p-value by the number of tests belonging to the same family and clipping values at 1.0. For the motion parameters, 20 tests were performed. Post-hoc tests were adjusted for multiple comparisons via Tukey’s HSD, ensuring the overall Type I error rate remained at 5%.

For the four post-experiment questionnaires (Qn1 in Fig. 2B), a two-way ANOVA with the same main effects (partner and instruction) was used. The subjective experience ratings after each trial (e.g., sympathy) were analyzed using a one-way ANOVA with four levels to test whether the belief about the interaction partner affected the rating. The four levels resemble veridically human, veridically artificial, falsely human and falsely artificial (Fig. 2C). The normality and homogeneity assumptions were met. To test whether ratings were reciprocated by the humans, we employed a repeated measure correlation between partners and adjusted for multiple comparisons. If detection metrics for the Turing test (FNR, FPR, accuracy) differed from a random prediction, we used Wilcoxon signed rank tests and tested against the chance-level performance of 0.5. In addition, we tested whether the duration of a trial influences the detection accuracy by employing a Mann–Whitney U test between the long- and short-term performance metric, again adjusting the p-value using the Bonferroni method. Dynamics over time, that is, a non-zero slope (Fig. 2A,D), were tested by fitting a linear regression and reporting its p-value based on the Wald test. To assess whether the detection accuracy is significantly different from a random guess (Table 1), we tested against a random guess (FNR/FPR of 50%) using the Wilcoxon signed-rank test.

#### Linear mixed-effects model

To assess the influence of motion parameters on the detection accuracy of the artificial agent, we employed a linear mixed-effects models using Pymer4, the Python wrapper for the Lmer R package. Pymer4 generalizes to binominal variables and allowed us to model nested random effects. We modeled the decision error $\varepsilon$ as a dependent variable (0 for correct, 1 for wrong). We included random effects for trial number and nested random effects for pairs of participants and individual participants. A random intercept for each condition was added to allow for variation not explained by the fixed effect. Motion parameters and the condition are fixed effects. The model was formalized as: $\varepsilon \sim \bar{v} + \bar{a} + \Delta \bar{v} + \Delta \bar{a} + d + \text {condition} + (1|\text {pair}/\text {participant}) + (1|\text {trial}) + (1|\text {condition})$.

#### Analyzing sensorimotor patterns

The follow-net was trained on motion data from the training session in order to learn patterns of human arm movements. To probe the patterns that the network observed in the training data, we calculated the input feature importance. In our scenario, this is the influence of each joint of the leader on every joint of the follower. We calculated the feature importance as the geodesic distance between the unit quaternions of the predictions on two nearly identical input sequences *s* and $s'$. The sequence $s'$ is a copy of *s*, but in $s'$, a certain joint (e.g., the head) was frozen to a random different pose, also sampled from this experiment. Since the sequences are slightly different, predicting on both sequences leads to different poses *q* and $q'$. The geodesic distance is the length of the shortest path between these two quaternions:

$$d(q, q') = \left| \log \frac{q'}{q}\right|$$

A larger value indicates that the substituted joint is more important for generating human-like arm movements.

### Questionnaires and ratings

Before commencing the experiment, we inquired the participants about their personality using the following questionnaires: AQ-short for autistic behavior^28^, SPF^29^ for the different domains of empathy, and Neo-FFI^30^ for the Big Five items (openness, conscientiousness, extraversion, agreeableness and neuroticism). Additionally, we asked about current physical well-being, i.e., about hours of sleep in the night before, how fit participants felt and whether they had eaten enough. This information however was not used in the current analysis.

During the experiment, after each trial, participants were asked to evaluate different aspects of their most recent subjective experience. They rated their own focus level, the synchronicity and creativity of movements and the sympathy towards the interaction partner. In the Turing test, they also decided whether the interaction partner was human or artificial and how confident they were in this decision.

Finally, there was a post-experiment interview about the experience during the experiment. We asked general questions about how well participants thought the artificial agent and the human partner had performed in the different conditions, about the perceived frequency of being in the role of the follower or the leader during improvisation, and about how often they thought to have answered correctly in the Turing test. Every post-experiment interview question that we analyzed was rated on an 11-point scale.

**Table 3:** Post-experiment interview questions.

	Question	Response Range
Qn1	How well did your partner perform? (respectively for the human- and artificial agent in every condition)	From bad (‘0’) to good (‘10’)
Qn2	How often did you interact with the agent/human?	From always artificial agent (‘0’) to always human agent (‘10’)
Qn3	During improvisation, how much of the time have you been the leader/follower? (respectively for human- and artificial agent)	From always follower (‘0’) to always leader (‘10’)
Qn4	Did improvisation predominantly consist of follow/lead change or co-leadership?	follow/lead (‘0’), co-leadership (‘10’)

## Data Availability

The datasets generated during and/or analyzed during the current study are available from the corresponding author on reasonable request.
